# Modulation of neural firing through intracellular ATP dynamics governed by energy feedback from the vascular system

**DOI:** 10.1186/1471-2202-16-S1-P26

**Published:** 2015-12-18

**Authors:** Karishma Chhabria, V Srinivasa Chakravarthy

**Affiliations:** 1 Department of Biotechnology, Indian Institute of Technology Madras, Chennai, 600036, Tamil Nadu, India

## 

We propose a simple model for neuro-glio-vascular interactions to emphasize on the role of energy feedback from the vascular system in brain's computations [1,2]. In [1], we introduced a bidirectional communication within a detailed biophysical model of neuron-astrocyte-vessel. We now compress this model to just two modules: the neuron and the 'energy' module. The energy module is a lumped representation of the astrocyte-vessel system; it receives neural firing activity as input and controls intracellular neuronal energy (ATPi) levels as a feedback. The model comprises of a quadratic integrate and fire neuron with a dynamic threshold, *V_th_*, which further depends on the ATPi dynamics. *V_th _*is high during ATPi deficit, making the neuron least excitable and vice versa for high ATPi conditions. The underlying principle of modeling *V_th _*as a function of ATPi is based on the experiments describing the role of KATP channels in governing neural excitability [3]. These channels are ATP-dependent potassium channels and are open when ATPi is low, resulting in a depolarized membrane potential of the neuron during metabolically compromised states such as hypoxia [3].

**Figure 1:**
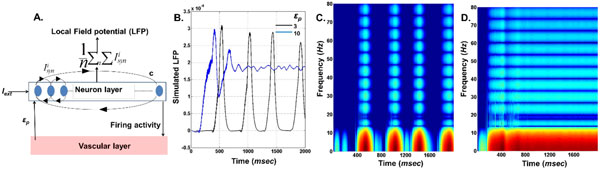
**A. Implemented network configuration with *n (=1000) *neurons, *i *synapses with *c (=20%) *connectivity**. **B**. Simulated LFP at both high (=10) and low (=3) *ε_p. _*C. & D. depicts spectrograms for low and high *ε_p_*, respectively.

The neuron model parameters are adapted to that of mammalian cortical pyramidal neuron [4]. Furthermore, ATPi dynamics are also modeled similar to [4], where ATPi consumption directly depends on neural spiking activity. The production rate of ATPi, *ε*_*p *_is a crucial model parameter representing the local vascular activity. A wide range of neural dynamic behaviors: phasic bursting, tonic bursting and continuous spiking are observed by varying *ε*_*p *_and external input current Iext. Furthermore, simulation of a network consisting of such energy-dependent neural units (Figure 1A.) depicts that *ε*_*p *_could modulate the Local field potential (LFP) frequencies and amplitudes (Figure 1B.). Interestingly, low frequency LFP dominates under low *ε*_*p *_conditions and could represent seizure-like activity observed in epilepsy. Although conventional neuroscience considers unidirectional influences from neurons to small vessels, there have been proposals that highlight the reverse influence from the vessels to the neurons [1,2] . The proposed 'neuron-energy' unit may be treated as a building-block in large scale models of neurovascular networks.
